# Effect of regioisomers of hydroxystearic acids as peroxisomal proliferator‐activated receptor agonists to boost the anti‐ageing potential of retinoids

**DOI:** 10.1111/ics.12730

**Published:** 2021-09-05

**Authors:** Anthony V. Rawlings, Eliane Wandeler, Igor Bendik, Pascale Fuchs, Jean‐Marc Monneuse, Dominik Imfeld, Rolf Schütz

**Affiliations:** ^1^ AVR Consulting Ltd. Northwich UK; ^2^ DSM Nutritional Products Ltd. Kaiseraugst Switzerland; ^3^ Phylogene SA Bernis France

**Keywords:** collagen, hydroxystearic acid, PPAR, proteomics, skin physiology/structure

## Abstract

**Introduction:**

We report on the in vitro and ex vivo effects of chiral (*R*)‐10‐hydroxystearic acid (10‐HSA) compared with other mono‐hydroxystearic acid regioisomers and stearic acid (SA) together with its benefit when combined with retinol.

**Methods:**

Following treatment with hydroxystearic acids peroxisomal proliferator‐activated receptor alpha (PPARα) activity was determined in a luciferase reporter gene assay, collagen type I was assessed in primary human dermal fibroblasts by immunohistochemistry, modification of the intracellular fibroblast collagen proteome was studied by mass‐spectrometry‐based proteomics and collagen type III was assessed by immunohistochemistry on human ex vivo skin.

**Results:**

10‐HSA was the most effective PPARα agonist (15.7× induction; *p *< 0.001), followed by 9‐HSA (10.1× induction) and then 12‐HSA (4.9× induction) with 17‐HSA (1.7× induction) being similar to the effects of stearic acid (1.8× induction). Collagen type I levels were increased in primary human fibroblasts by 2.12× and 1.56× for 10‐HSA and 9‐HSA, respectively, in vitro with the10‐HSA being significant (*p *< 0.05), whereas 12‐HSA and SA had no statistical effect over the untreated control. 10‐HSA and 12‐HSA modified the intracellular fibroblast collagen proteome slightly with significant increases in collagen alpha‐1 (VI) and alpha‐3 (VI) proteins but only 10‐HSA increased levels of collagen alpha‐2 (V), alpha‐1 (III), alpha‐1 (I) and alpha‐2 (I) (all *p *< 0.05) with the increases being significantly different between 10‐HSA and 12‐HSA for collagen alpha‐1 (I), collagen‐3 (VI) and collagen alpha‐2 (I) (*p *< 0.01). Collagen type III in ex vivo skin was increased +47% (*p *< 0.05) by 0.05% (1.7 mM) retinol, +70% (*p *< 0.01) by 0.01% (0.33 mM) 10‐HSA and the combination increased levels by +240% (*p *< 0.01 for either ingredient).

**Conclusion:**

Chiral (*R*)‐10‐HSA has been shown to be superior to 9, 12 and 17‐HSA as a PPARα agonist. Moreover, 10‐HSA stimulated collagen synthesis in monolayer fibroblast culture as assessed by proteomics and immunohistochemically. Furthermore, we also show the synergistic effects of 10‐HSA with retinol on collagen III synthesis in skin explants. These results further highlight the efficacy of 10‐HSA as a cosmetically acceptable PPARα agonist and anti‐ageing ingredient.

## Graphical abstract

Chiral (*R*)‐10‐HSA has been shown to be superior to 9‐HSA, 12‐HSA and 17‐HSA as a PPARα agonist. Moreover, 10‐HSA stimulated collagen synthesis in monolayer fibroblast culture as assessed by proteomics and immunohistochemically. Furthermore, we also show the synergistic effects of 10‐HSA with retinol on collagen III synthesis in skin explants highlighting the efficacy of 10‐HSA as a cosmetically acceptable PPARα agonist and anti‐ageing ingredient.

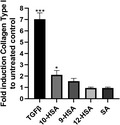

## Abstrait

**Introduction:**

Nous rapportons les effets in vitro et ex vivo de l'acide chiral (R)‐10‐hydroxystéarique (10‐HSA) par rapport à d'autres régioisomères d'acide mono‐hydroxystéarique et à l'acide stéarique (SA) ainsi que ses avantages lorsqu'il est associé au rétinol.

**Méthodes:**

Après un traitement avec des acides hydroxystéariques, l'activité du récepteur alpha activé par les proliférateurs peroxysomaux (PPARα) a été déterminée dans un test du gène rapporteur de la luciférase, le collagène de type I a été évalué dans les fibroblastes dermiques humains primaires par immunohistochimie, la modification du protéome du collagène des fibroblastes intracellulaires a été étudiée par spectrométrie de masse. La protéomique et le collagène de type III ont été évalués par immunohistochimie sur la peau humaine ex vivo.

**Résultats:**

la 10‐HSA était l'agoniste PPARα le plus efficace (induction 15,7X ; p<0,001), suivi de la 9‐HSA (induction 10,1X) puis de la 12‐HSA (induction 4,9X) avec la 17‐HSA (induction 1,7X) étant similaire aux effets de l'acide stéarique (induction 1,8X). Les niveaux de collagène de type I ont été augmentés dans les fibroblastes humains primaires de 2,12X et 1,56X pour la 10‐HSA et la 9‐HSA respectivement in vitro, la 10‐HSA étant significative (p<0,05) : alors que la 12‐HSA et la SA n'ont eu aucun effet statistique sur le témoin non traité. La 10‐HSA et la 12‐HSA ont légèrement modifié le protéome du collagène des fibroblastes intracellulaires avec des augmentations significatives des protéines de collagène alpha‐1 (VI) et alpha‐3 (VI), mais seule la 10‐HSA a augmenté les niveaux de collagène alpha‐2 (V), alpha ‐1 (III), alpha‐1 (I) et alpha‐2 (I) (tous p<0,05) avec des augmentations significativement différentes entre 10‐HSA et 12‐HSA pour le collagène alpha‐1 (I), le collagène‐ 3 (VI) et Collagène alpha‐2 (I) (p<0,01). Le collagène de type III dans la peau ex vivo a augmenté de +47 % (p<0,05) de 0,05 % (1,7 mM) de rétinol, de +70 % (p<0,01) de 0,01 % (0,33 mM) de 10‐HSA et la combinaison a augmenté les niveaux de +240 % (p<0,01 pour chaque ingrédient).

**Conclusion:**

La chiral (R)‐10‐HSA s'est avérée supérieure à 9, 12 et 17‐HSA en tant qu'agoniste de PPARα. De plus, la 10‐HSA a stimulé la synthèse de collagène dans la culture de fibroblastes monocouche telle qu'évaluée par protéomique et immunohistochimique. De plus, nous montrons également les effets synergiques de la 10‐HSA avec le rétinol sur la synthèse du collagène III dans les explants de peau. Ces résultats soulignent en outre l'efficacité de la 10‐HSA en tant qu'agoniste de PPARα et ingrédient anti‐âge cosmétiquement acceptable.

## INTRODUCTION

We have previously published the effect of the peroxisomal proliferator‐activated receptor (PPAR) agonist, 10‐hydroxystearic acid (10‐HSA), as a novel anti‐ageing ingredient that particularly reduces the appearance of pores and hyperpigmented lesions [1]. As reduced type I and III collagens are reported in both chronologically aged and in photodamaged skin, the biochemical induction of fibroblast collagens type I and III with 10‐HSA indicated its potential skin anti‐ageing effects [1, 2, 3, 4]. Obviously, improvements in the overall skin ageing process are not limited to the induction of collagen proteins only. For example, the effects of tretinoin on the elastin and fibrillin‐rich oxytalan fibres are well established [4]. However, 10‐HSA is also known to induce and secrete other important extracellular matrix proteins in fibroblasts *in vitro* [1].

Effects of other hydroxystearic acid variants are reported in the literature [5, 6], and one, 12‐HSA, has also been identified as a PPARα agonist and has been used topically [7, 8]. However, the relative efficacies of these two hydroxystearic acid isomers as PPARα agonists remain unreported as well as the phenotypic response they induce on fibroblast collagen synthesis. We also wanted to evaluate the structure–activity relationship of mono‐hydroxy stearic acids that are naturally occurring or easy to produce such as from saturated fatty acids, either by enzymatic or by chemical synthesis. As a result, we report the efficacy of 9‐HSA, 10‐HSA, 12‐HSA and 17‐HSA together with stearic acid.

Moreover, retinoids are also used as anti‐ageing ingredients that also induce collagen synthesis with type III collagen induction being more promoted by retinoic acid (RA) compared with retinol (ROH) [9]. As a result, the use of type III collagen as an RA‐responsive marker maybe useful to identify ROH boosting strategies. Co‐treatment of retinoids (retinoic acid or retinyl retinoate) with PPARα agonists has been reported to reduce retinoid dermatitis suggesting benefits from combining agonists to the retinoid and PPAR transcription factors [10]. This could be the result of activating retinoid and PPAR pathways independently or may be the result of PPARα interacting with the retinoid X receptor (RXR) [11]. As a result, the additional focus of this preliminary work was to also demonstrate the efficacy of ROH combined with 10‐HSA on collagen synthesis *ex vivo*.

## MATERIALS AND METHODS

### Hydroxystearic acid variants

10‐HSA was synthesized as previously described [1]. 9‐HSA and 17‐HSA were synthesized using a proprietary method. 12‐HSA and SA were purchased from Sigma‐Aldrich, Buchs, Switzerland. All variants were at least 95% pure.

### Experimental strategy

To determine the effectiveness of HSA isomers as PPARα agonists, analyse the effects of 10‐HSA and 12‐HSA on extracellular matrix marker production (collagen type I) in fibroblast monolayer cultures, compare the collagen proteome by mass spectrometry proteomics and evaluate the synergistic response of 10‐HSA together with retinol (ROH) on collagen type III synthesis in skin explants. All methods are similar to that published previously with the exception that the intracellular fibroblast collagen proteome is reported [1].

### PPARα transactivation assay

A one‐hybrid system was applied that used a hybrid construct of GAL4 DNA‐binding part coupled to the human PPARα ligand‐binding domain, a luciferase reporter plasmid and a renilla‐expressing plasmid to normalize transfection efficiency. For transient transfections, white 96‐well cell culture plates with clear bottom (Corning, Basel, Switzerland) were used. 7.5 × 104 HEK293 cells per well were plated in minimum essential medium (Eagle) at 37°C in 5% CO_2_ without phenol red supplemented with 10% charcoal‐treated foetal bovine serum (HyClone Laboratories, Inc., Logan, UT, USA). The cells were transiently transfected at 70%–80% confluence by polyethylene‐mine‐based transfection for 5 h at 37°C, 5% CO_2_, which was followed by respective applications of the test compounds (1.7 µM) dissolved in DMSO as a vehicle (0.45% final DMSO concentration was achieved in the wells). The GW7647 (1 µM) compound was used as a reference for human PPARα. Stimulations lasted 16 h according to established protocols. All plates of a campaign were treated the same, and the only difference was the stimulation by the different compounds All compounds were tested as 4 replicates with the exception of the vehicle (*n* = 52) and positive (*n* = 24) controls. The measurement of firefly and renilla luciferase luminescence was conducted sequentially in the same cell extract using buffers according to established protocols (Promega AG, Dübendorf, Switzerland). Relative transactivation values were calculated as the ratio of the luminescence of the firefly to renilla results. IBM SPSS software version 22 (SPSS; Chicago, IL, USA) was used for all statistical analysis. For multiple comparison of the means, we applied one‐way ANOVA that was followed by Bonferroni multiple comparisons post hoc test with a significance level of *p *< 0.05.

## MEASUREMENT OF TOTAL CELLULAR COLLAGEN TYPE I CONTENTS IN NORMAL HUMAN FIBROBLASTS

Human dermal fibroblasts obtained from adult skin were seeded into 96‐well plates (4000 cells/well) and cultured in Dulbecco's Modified Eagle's Medium high glucose (DMEM; Gibco Invitrogen, Basel, Switzerland) containing 10% foetal calf serum (FCS; Amimed BioConcept, Allschwil, Switzerland) and 1% penicillin/streptomycin (P/S; Invitrogen, Switzerland) at 37°C with 5% CO_2_ for 24 h. Subsequently, the cells were starved in DMEM low glucose containing 0.2% FCS and 1% P/S for 2.5 days. Then, the starvation medium was refreshed together with the addition of the solubilized test compounds and incubated for another 48 h. Thereafter, HSA (5 µM) was diluted in the culture medium from a 10 mM dimethylsulphoxide (DMSO) stock solution. Transforming growth factor‐beta 1 (TGF‐β1; PeproTech, Hamburg, Germany) was used as positive control (10 ng/ml). After compound incubation cells were fixed in Dulbecco's phosphate‐buffered saline (DPBS; Gibco Invitrogen, Switzerland) containing 4% formaldehyde (Life Technologies, Zug, Switzerland) for 15 min and permeabilized with 0.1% Triton‐X100 (Sigma‐Aldrich, Buchs, Switzerland) in DPBS for 90 s. Collagen type I or III were detected using mouse anti‐human collagen I antibody (Millipore, Switzerland) and rabbit anti‐human type III collagen (BioTrend, Cologne, Germany), respectively, followed by a AlexaFluor 488‐conjugated secondary antibody (goat anti‐mouse or anti‐rabbit IgG; Life Technologies, Zug, Switzerland). We counterstained the nuclei with 4′,6‐diamidino‐2‐phenylindole (DAPI; Sigma‐Aldrich, Buchs, Switzerland). Image acquisition and quantitative analysis were performed using an ArrayScan^®^ VTI HCS imaging system (Thermo Scientific, Waltham, MA, USA) with 49 pictures per well with 10× objective. Collagen was measured intracellularly, and the fluorescence intensity values were normalized to the cell count. Data are based on a minimum of three independent measurements and were represented as mean values ± standard deviations (SD); differences between groups were evaluated by one‐way ANOVA test followed by pairwise post hoc comparisons Dunnett's permutation test to the media control sample with a significance level of *p *< 0.05.

### Fibroblast collagen proteomics following treatment with 10‐HSA and 12‐HSA

Human dermal fibroblasts (HDF) isolated from a biopsy of an adult female donor (Fb‐D) were maintained in Dulbecco's modified Eagle's Medium high glucose (DMEM) containing 10% foetal calf serum (FCS) and 1% penicillin/streptomycin (P/S). Cells were cultivated at 37°C in a humidified 5% CO_2_‐air atmosphere. HDF were seeded in 6‐well plates (120’000 cells per well) and cultured for 24 h in DMEM 10% FCS 1% P/S before being starved in DMEM low glucose containing 0.2% FCS and 1% P/S for 2.5 days. Starvation medium was then replaced, and solubilized compounds (5 µM) were added and incubated for 48 h. The preparation of the intracellular proteins from the treated cells was performed using 20 mM ammonium hydroxide and stringent washing with water. Intracellular protein isolates obtained from the cells were dissolved in 600 µl of 50 mM ammonium bicarbonate buffer containing 0.5 µg of trypsin was added in each culture plate well. Plates were incubated 1.5 h at 37°C. Total protein content was determined using the BCA kit from Thermo Scientific (Illkirch, France). Lysates from the same sample were pooled and acidified to block trypsin activity. Lysates were evaporated to dryness (SpeedVac, Thermo Scientific, France). Proteins were then reduced (DTT) and alkylated (IAA) and digested a second time with trypsin to complete proteins digestion. For all samples, peptides were purified by SPE chromatography (C18), dried and solubilized in 100 µl of 0.1% formic acid aqueous solution. Peptides digests (500 ng per run) were analysed by Eksigent Ultra Plus nano‐LC 2D HPLC coupled to a TripleTOF 5600 (AB Sciex, Framingham, USA) mass spectrometer interfaced to a nano‐spray III source. The absolute signal of peptide or protein was calculated by summing the extracted area of all unique fragment ions. For *p*‐value calculation, a bilateral *t* test for homoscedastic variance was applied. *p *< 0.05 was considered to be statistically significant.

### Ex vivo skin sampling and type III collagen quantification

Human skin from abdominal plastic surgery was obtained from healthy Caucasian donors after their informed consent and was used for the analysis of collagen type III. Skin samples were cut to 8 × 3 mm (diameter × thickness) weighed and, if necessary, reduced in the dermal portion, to have approximately the same weight. They were maintained in an air‐liquid interface in contact with culture medium (modified Williams’ E medium, Thermo Fisher Scientific, Waltham, MA, USA) up to 6 days. Six skin samples for each treatment were cultured to perform the collagen assay and two samples for viability test. After 6 days of incubation and treatment, two samples per condition were processed with methylthiazolyldiphenyl‐tetrazolium bromide (MTT) according to supplier's instructions (Roche Applied Science, Rotkreuz, Switzerland). Skin viability was measured with a plate reader at a wavelength of 570 nm. From each of the six skin samples, two sections (*n* = 12) were immunostained with monoclonal mouse anti‐collagen III (Sigma‐Aldrich, cat#c7805, Buchs, Switzerland) and the alkaline phosphatase/RED detection system (Dako #K5005; Agilent Technologies, Glostrup, Denmark). The amount of the antigen present in each slide was graded by the pixel intensity and the distribution of the red staining within a selected area of the upper part of the dermis using ImageJ (NIH; Bethesda; MD, USA) resulting in a staining score which was then normalized as a percentage of the untreated control. All quantitative data were summarized in terms of the mean score and standard error of mean (SEM) for each treatment. Differences between groups were evaluated by one‐way ANOVA with permutation test followed by pairwise post hoc comparisons Dunnett's permutation test and pairwise post hoc comparisons Tukey's HSD permutation tests.

## RESULTS AND DISCUSSION

In this series of studies, we compared the relative PPARα agonistic activities of hydroxystearic acids together with other selected bioactive lipids using the reporter gene assay in HEK293 cells published previously [1]. As can be seen from Table 1 when tested at 1.7 µM, 10‐HSA was the most effective agonist, followed by 9‐HSA and then 12‐HSA. When combined together 9‐HSA and 10‐HSA were of intermediary value to their parent acids (data not shown). Moreover, there was significant reduction in PPARα transactivating activity with 17‐HSA compared with 9‐HSA, 10‐HSA and 12‐HSA and was similar to the effects of stearic acid. Whereas 12‐HSA possessed only 2.6 times the PPARα agonist activity of stearic acid, 10‐HSA has 8.5 times the activity. Overall, 10‐HSA was 3.2 times more effective as a PPARα agonist than 12‐HSA (*p *< 0.001). This highlights the importance of the hydroxy position on the stearate backbone with the 17‐hydroxy positional isomer having minimal PPAR activity and very similar to that of stearic acid, and 9‐HSA having approximately 66% activity of 10‐HSA, while 12‐HSA had only 30% activity. Interestingly, Yokoi et al. [5] have also reported the optimal PPARα reporter activity of 10‐hydroxyoctadecenoic acid compared with 8, 11 and 13 positional isomers. Thus, it appears that the positional isomerism of the hydroxy group on these acids has a major effect on their PPARα agonistic activity similar to that reported for hydroxystearic acid isomers. Other conventional fatty acids possessed low agonist activity (<2.8‐fold), in the order docosahexaenoic acid > petroselinic acid = linoleic acid > stearic acid > arachidonic (data not shown). Our PPARα one‐hybrid test might be possible in HaCaTs or immortalized fibroblasts. However, we have never tested, nor established nor validated such assays. We believe that our established and validated PPARα one‐hybrid assay in HEK cells is sufficiently suited to demonstrate transactivation of PPARα receptors and that the agonistic findings of the HSAs on PPARα receptors are also transferable to keratinocytes and fibroblasts, as they express the PPARα receptors.

**TABLE 1:** Fold induction of PPARα by different SA variants tested at 1.7 µM

Compound	Transactivation [normalized luminescence values]		N	Fold change (vs vehicle)	*p*‐values (ANOVA) (Post hoc Bonferroni)**					
	Means	SEM*			Vehicle	10‐HSA	9‐HSA	12‐HSA	17‐HSA	Stearic acid
Vehicle (DMSO)	0.370	0.0070	52			<0.001	<0.001	<0.001	1	1
GW 7647 (1 µM)	3.319	0.1054	24	9.0	<0.001	<0.001	1	<0.001	<0.001	<0.001
10‐Hydroxystearic acid (1.7 µM)	5.810	0.3680	4	15.7	<0.001		<0.001	<0.001	<0.001	<0.001
9‐Hydroxystearic acid (1.7 µM)	3.739	0.2619	4	10.1	<0.001	<0.001		<0.001	<0.001	<0.001
12‐Hydroxystearic acid (1.7 µM)	1.797	0.2591	4	4.9	<0.001	<0.001	<0.001		<0.001	<0.001
17‐Hydroxystearic acid (1.7 µM)	0.623	0.0336	4	1.7	1	<0.001	<0.001	<0.001		1
Stearic acid (1.7 µM)	0.684	0.0361	4	1.8	1	<0.001	<0.001	<0.001	1	

* SEM Standard error of the mean

** ANOVA used Bonferroni post hoc test for multiple comparison with a significance level of 0.05. *p* = 1 not significant.

**FIGURE 1:**
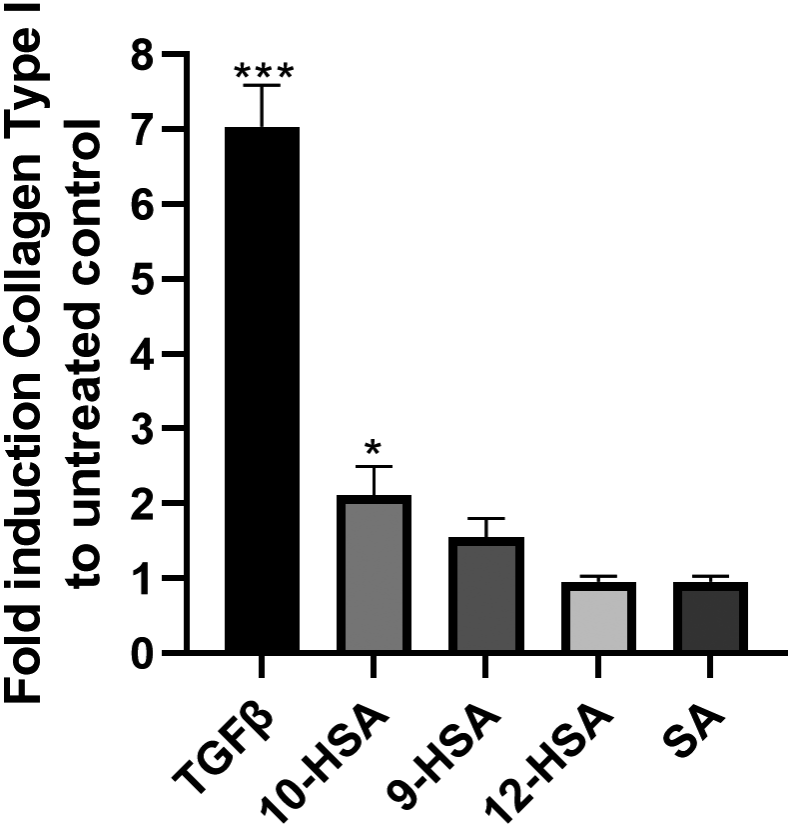
Fold induction of collagen type I in fibroblast monolayer cultures by SA variants (5 µM). Post hoc comparisons using Dunnett's test indicated TGFβ (10 ng/ml) was significantly different to untreated control (*p *< 0.001***) as well as 10‐HSA (*p *< 0.05*)

Transactivation of PPARα in a reporter gene assay does not guarantee a cellular response in a PPARα response gene as recruitment of coactivators or release of corepressors is not evaluated. As a result, demonstration of a marker protein is essential in the cell type of interest. We have previously demonstrated the type I and III collagen inducing activity of 10‐HSA in fibroblast monolayer cultures and in skin explants [1]. Here, we compared the relative efficacy of 12‐HSA, 9‐HSA and stearic acid at inducing type I collagen in fibroblast monolayer cultures (all at 5 µM). 10‐HSA was shown to be numerically superior to the other acids tested and significantly increased collagen type I expression relative to the untreated control (Figure 1, *p *< 0.05). 9‐HSA responded similarly to that observed in the reporter gene assay compared with 10‐HSA but not being significant to media control. Surprisingly, 12‐HSA and stearic acid were not effective at all. This is very peculiar for 12‐HSA, especially as it is reported to be a PPAR agonist [7, 8].

Proteomic analysis of fibroblast cellular proteins also demonstrated differences in collagen synthesis following 10‐HSA and 12‐HSA (5 µM) treatment (Table 2). Induction of collagen alpha‐1 (I) and collagen alpha‐2 (I) following treatment with 10‐HSA, but not 12‐HSA was confirmed. 10‐HSA also increased the levels of collagen alpha‐2 (V) and collagen alpha‐1 (III). Both acids increased the levels of collagen alpha‐1 (VI) and collagen alpha‐3 (VI), but 10‐HSA induced their levels more greatly. The increases in collagen peptides were significantly greater for 10‐HSA compared with 12‐HSA for collagen alpha‐1 (I), collagen‐3 (VI) and collagen alpha‐2 (I) (*p *< 0.01). Although these changes are small, these results are consistent with the immunohistochemistry of the fibroblast monolayer culture experiments.

**TABLE 2:** Fold change of collagen proteins induced by 10‐HSA and 12‐HSA (5 µM) in primary fibroblast monolayer cultures determines by mass spectrometry proteomics

Protein	10‐HSA Fold change	*p*‐value 10‐HSA to vehicle	12‐HSA Fold change	*p*‐value 12‐HSA to vehicle	*p*‐value 10‐HSA to 12‐HSA
Collagen alpha‐2 (V)	1.35	*p *< 0.05	1.19	NS	NS
Collagen alpha‐1 (III)	1.25	*p *< 0.001	1.03	NS	NS
Collagen alpha‐1 (I)	1.16	*p *< 0.01	0.94	NS	*p *< 0.001
Collagen alpha‐1 (VI)	1.14	*p *< 0.01	1.06	*p *< 0.01	NS
Collagen alpha‐3 (VI)	1.12	*p *< 0.001	1.07	*p *< 0.001	*p *< 0.01
Collagen alpha‐2 (I)	1.11	*p *< 0.05	0.97	NS	*p *< 0.001

**FIGURE 2:**
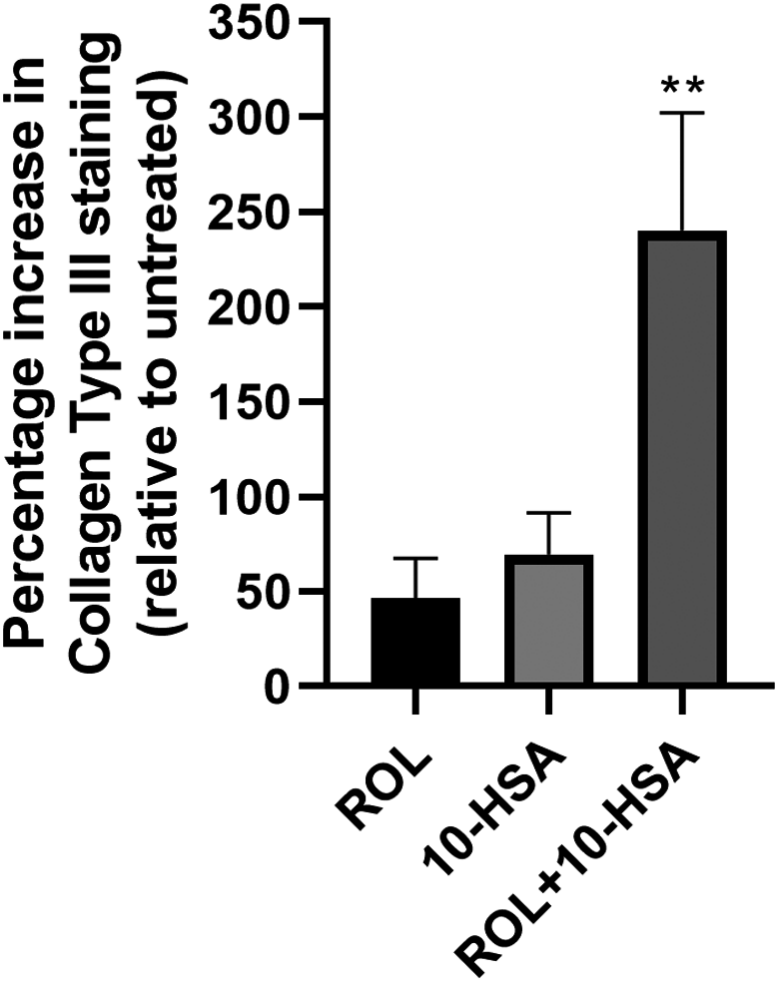
Induction of collagen type III in skin explants by retinol (0.05%), 10‐HSA (0.01%) and the combination. The combination was significantly different to the individual compounds (*p *< 0.01**)

Boosting the anti‐ageing efficacy of ROH and/or its esters is of great interest topically. It is possible that this may be achieved with PPAR agonists. Moreover, synergy with PPAR agonists may offer routes to reduced skin irritation from retinoids. In this respect, a PPARα agonist (WY14643) has been shown to reduce retinoid skin irritation [10]. Here, we were interested in the retinol boosting activity on collagen type III protein expression when combined with 10‐HSA when applied topically to skin explants (Figure 2). Retinol (0.05%) and 10‐HSA (0.01%) increased the protein expression of collagen type III by +47% and +70% respectively, when combined the expression increased by +240% at 0.05% ROH and 0.01% 10‐HSA concentrations respectively. All tests were significantly different to the vehicle control (*p *< 0.05), and the combinations were also significantly different to the individual compounds (*p *< 0.05). Thus, 10‐HSA cooperates with retinol in collagen biosynthesis probably by 10‐HSA bound PPARα partnering with RXR like that reported in astrocytes [12], although we cannot exclude an RAR‐RXR response or both. Although fatty acids are also reported to bind to the retinoid X receptors, the transcriptional heterodimer partners involved in a PPAR response, hydroxy monounsaturated fatty acids do not [5, 11, 13]. Clearly, for the 10‐HSA and retinol synergistic response, retinol must be converted to other retinoids for effects on RAR and/or RXR and for their effects in skin [14, 15, 16]. It is possible that the enzyme machinery for retinoid synthesis has been upregulated by 10‐HSA bound PPARα as has been shown for PPARγ in human dendritic cells [17]. We are in the process of determining the precise biochemical mechanisms for the effect between ROH and 10‐HSA.

## CONCLUSIONS

In conclusion, chiral (*R*)‐10‐HSA has been shown to be superior to 9‐HSA, 12‐HSA and 17‐HSA as a PPARα agonist. Moreover, 10‐HSA stimulated collagen synthesis in monolayer fibroblast culture as assessed by proteomics and immunohistochemically. Furthermore, we also show the synergistic effects of 10‐HSA with retinol on collagen III synthesis in skin explants. These results further highlight the efficacy of 10‐HSA as a cosmetically acceptable PPARα agonist and anti‐ageing ingredient.
